# Effectiveness of a Nutrition Education Program to Improve Children's Chewing Habits

**DOI:** 10.1155/2016/4304265

**Published:** 2016-03-10

**Authors:** Nanae Sato, Fumi Hayashi, Nobuo Yoshiike

**Affiliations:** ^1^Department of Nutritional Sciences, The University of Morioka, 808 Sunakomi, Takizawa, Iwate 020-0694, Japan; ^2^Nutrition Ecology, Department of Nutrition Sciences, Kagawa Nutrition University, 3-9-21 Chiyoda, Sakado, Saitama 350-0288, Japan; ^3^Department of Health Sciences, Aomori University of Health and Welfare, 58-1 Mase, Hamadate, Aomori, Aomori 030-8505, Japan

## Abstract

This quasi-experimental study determined whether the nutrition education program we developed to promote chewing food properly influenced children's chewing habits successfully. Four kindergarten classes in Japan (150 children, aged 5-6 years) were studied; one class received the educational program in the classroom and at home (Group A) and three classes received the program in the classroom only (Group B). The educational program was integrated into the classes' daily curriculum for five weeks. It included storytelling with large picture books, chewing consciously while eating lunch, singing a song with gestures, and greetings before and after meals (both groups). Group A also used a paper textbook and was provided information by the leaflet to encourage guardians to implement the program at home. Chewing habits before and after intervention were evaluated: (1) guardians completed seven questionnaire items related to chewing habits and chewing movement and (2) the number of chews and time spent eating the test meal were measured by a portable chewing sensor. Both approaches improved the children's chewing habits; however, no difference was found between the two groups. We concluded that this intervention could be used to improve chewing habits in young children even without active involvement of their guardians.

## 1. Introduction

In Japan, the number of young children with problems related to chewing habits (does not chew properly, does not like to chew) or ability (cannot chew; chewing ability is not completely developed yet) is increasing [1]. Chewing habits and ability develop through eating experiences during the weaning period and early childhood [2]. Therefore, weaning practices that develop desirable chewing function are recommended [3]. Since Japanese infants complete deciduous dentition at around three years of age, the earliest adult-like chewing ability that can be acquired is after three years old [4]. Since this is the period when the body, mind, and senses grow, children begin to develop a positive attitude toward and interest/concern in eating [2]. Functionally, the palate is growing and self-sufficient; self-motivated eating becomes possible. In other words, this is an opportune time to develop desirable chewing habits through self-motivated eating experiences. At this time, if children feel a meal is fun, they enjoy chewing with relish that will help improve their chewing habits.

Chewing stimulates the ventromedial hypothalamic nucleus of the satiety center and activates the histaminergic system, which may reduce appetite [5]. In recent years, chewing food properly may lead to slower eating and reduced dietary intake; studies have focused on chewing habits and obesity or diabetes [6–13]. One observational study found that individuals' rate of eating was associated with body mass index (BMI) [6–8] as well as an increased risk of excess weight [9] and insulin resistance or diabetes [10, 11]. Other intervention studies have found that participants who ate more slowly consumed fewer total calories [12] and that dietary intake in overweight adolescents could be controlled through behavior modification toward slow eating, when aided by a device that measures eating speed [13]. Based on this, as part of a Japanese national program to control metabolic syndrome in adults, acquisition of good chewing habits (chewing properly and eating slowly) has been introduced as a behavior modification technique to prevent overeating and reduce obesity [14]. Therefore, from the viewpoint of timely prevention to reduce the risk of future obesity and lifestyle-related diseases, it may be effective to begin intervention related to acquisition of good chewing habits as early as possible.

In addition, the subject of chewing habits was included for the first time in the second Basic Program for Nutrition Education Promotion announced in March 2011. Since then, nutrition education regarding chewing habits for younger children has been furthered through classroom instruction in educational institutions like kindergartens and schools [15].

However, the effectiveness of education programs has not been evaluated sufficiently and programs themselves appear to vary widely in content. Therefore, there may be a need to design and implement nutrition education programs that are effective and easily adopted.

Most of the empirical studies of the effectiveness of nutritional education concerning chewing habits in groups of young children are quantitative assessments of chewing* ability* [16–18]. There have been no quantitative studies to determine the effectiveness of the chewing* habits* modification outcomes of nutritional education programs focused on the chewing habits of young children.

Thus, for this study, we designed a nutritional education program that could be introduced into kindergartens to encourage “try to chew properly while enjoying eating”; we quantitatively assessed children's chewing habits to evaluate program effects. Because guardian involvement is important in children's behavior modification in early childhood [19], we established two program groups, one including the guardians and one without; we hypothesized that chewing habits would be more significant for children in the group receiving the intervention at home and in the classroom compared to the children who only received the intervention in the classroom.

## 2. Methods

### 2.1. Study Design

The study was structured following a quasi-experimental design.

### 2.2. Participants and Setting

**Figure 1:**
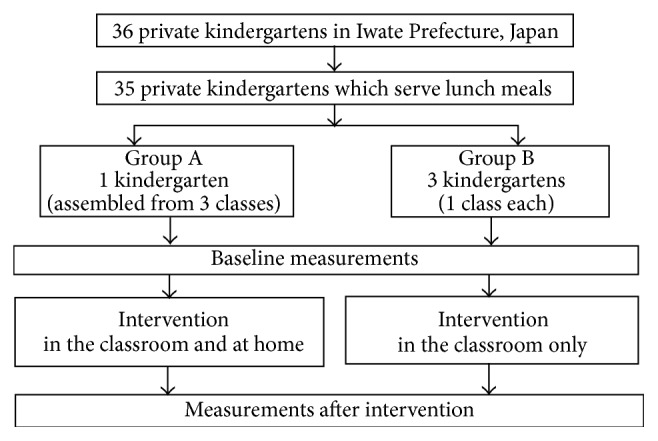
Study design.

Figure 1 depicts the study design. Participants were 5-6-year-old children attending kindergarten. Intervention sites were selected from among 36 kindergartens located in and around the city of Morioka in Iwate Prefecture, Japan. First, we identified 35 kindergartens that provided school lunch so we could control the test meal menu and thus enable repeated measurements of the number of chews, both before and after intervention. We then examined the number of classes and the number of children per class across kindergartens and selected a final group of four kindergartens for this study. Three classes (81 children) from one kindergarten were assigned to the group in which the educational program was conducted at home (Group A) and one class from each of three kindergartens (69 children) was assigned to the group in which the educational program was conducted in the classroom only (Group B).

### 2.3. Intervention

Table 1 shows the educational program as implemented within the kindergarten classes' daily activities for five weeks. We developed this program consistent with the items and aims presented in the “Curriculum Guidelines for Kindergartens” developed by the Ministry of Education, Culture, Sports, Science and Technology.

Before the program was initiated, a pretest was administered to a sample of 20 guardians from one kindergarten who each had a child (not included in the study) the same age as the participants. The educational program was then revised in terms of frequency of practice.

#### 2.3.1. In-Classroom Components (Groups A and B)

Both groups performed the following program activities in the classroom: storytelling with large picture books (once a week); greetings before and after a meal (every day); singing in chorus with filksong and hand gestures (three times a week); and chewing consciously while eating lunch (once a week).

#### 2.3.2. In-Classroom and At-Home Components (Group A Only)

We developed a nutrition education paper textbook for Group A. This paper textbook comprised eighteen color pages and merchandise with lovely original characters; it included a message to the guardian and a description of the program aim and classroom activities. It also presented useful information about conducting the program at home, which was checking of the child's daily eating habits by the guardian. We provided the paper textbook to each child.

Group A's program activities included daily use of the paper textbook by both the classroom teacher and the guardians (daily) and providing information to encourage guardians to do the program at home (once a week).

#### 2.3.3. Intervention Period and Advance Preparation

The intervention was conducted in each class over five weeks after summer vacation (August 27 to September 25, 2012). We conducted two training sessions with the class teachers of each kindergarten prior to the initiation of the intervention in order to ensure that the program would be delivered consistently. During these sessions, we demonstrated the standard instruction protocol and the checklist to confirm that the protocol was followed. We also presented the study outline and method to the teachers. The teachers in both groups encouraged the children and confirmed that the children performed the necessary activities. The class teachers in Group A also used the nutrition education paper textbook, requiring the children to bring the paper textbooks home and then collecting them at the study conclusion. The teachers in Group A distributed leaflets of information about the protocol to their students' guardians.

### 2.4. Measurements

Our results were compiled from a combination of measurement techniques previously identified as useful to evaluate chewing habits in children [20–22]: (1) subjective evaluation by the guardians using a questionnaire survey and (2) objective measurement of the children's chewing habits.

#### 2.4.1. Subjective Questionnaire Evaluation

We used a screening sheet called “Chewing Habit Chart” [23] to evaluate children's chewing habits; it is used in routine dental screenings conducted with all 3-year-olds. The criterion-related validity of the questionnaires was confirmed in that study. Before the survey was initiated, a pretest was conducted with the same 20 guardians as previously mentioned to check for distortions in the response distribution.

#### 2.4.2. Objective Evaluation Using the Measurement of Chewing Habits

The same test meal was prepared for all participating children in each kindergarten class. The “Kami Kami Sensor Measures Chews” (hereinafter, “sensor”) (Japan, Nitto Kagaku Co., Ltd.) was used to measure the number of chews and the time spent eating the test meal. This method is considered suitable to evaluate chewing habits in children [20, 22]. More detailed information about measurement using this sensor and the reproducibility of this method have previously been described. The time-adjusted number of chews (to account for the influence of meal duration and to provide an indicator for evaluating chewing habits) was calculated and defined as an objective evaluation index [22]: time-adjusted number of chews =* a* +* b*, where* a* is the residual for participants from the regression model (number of chews as the dependent variable and time spent eating the test meal as the independent variable) and* b* is the expected number of chews per person with the mean time spent eating the test meal.

### 2.5. Data Collection

#### 2.5.1. Questionnaire Survey

We administered the questionnaire before and after intervention by pretest-posttest design. The questionnaire was distributed to each student's guardian; the completed questionnaires were then collected in the classroom.

The questionnaire consisted of 15 items and measured four dimensions of chewing in children: (1) chewing habits “does not chew properly when eating”; (2) chewing movements “finishes a meal quickly and chew food roughly”; (3) chewing abilities to specific foods “cannot bite food (e.g., corn-on-the-cob, dried whole small fish) into smaller pieces”; (4) swallowing state “does not swallow food easily after putting it into the mouth”; the items were rated on a five-point Likert scale ranging from 1 (*always*) to 5 (*never*).

#### 2.5.2. Chewing Habits Measurement

Four dental hygienists were engaged to assist with the measurement process. They received training from one of our researchers on the measurement manual we prepared, which addressed the following: (1) the flow of the program and the dental hygienist's role, (2) basic concepts of and how to fit the sensor, (3) considerations for fitting and measurement, (4) how to handle a child who refuses to wear the sensor, and (5) training on sensor operation. Measurements were completed at each kindergarten by the same four dental hygienists on a predetermined day scheduled with the kindergarten. In addition, the researcher explained the measurement methods to all participants and kindergartens in order to diminish any variation in the training. Additionally, masking the measurement of the number of chews was impossible because of the nature of the procedure. Thus, we encouraged them to eat naturally and to be relaxed. The researcher demonstrated the measurement procedure to the participants, and then the sensor was fitted and checked by the researcher and the dental hygienists. Measurements began with whichever child that first finished the fitting. After initiating measurement, the children were observed while they ate; sensor fit and sensitivity were then checked. Technical problems that occurred during measurement were immediately addressed. If measurement accuracy did not improve after adjusting the sensor, the physical characteristics affecting the measurement fit and sensitivity (distance between and shape of the lower jaw and the throat) as well as the chewing characteristics of the child were observed and recorded. The sensor was stopped when the child finished eating. The number of chews and time required to finish the meal were recorded.

#### 2.5.3. Physical Status and Oral Conditions

Anthropometric measurement is performed once a month by the kindergartens. We obtained measurement data (body height and weight) from the kindergartens in June, the month before the initiation of the survey. We also obtained data from dental exams performed by the end of June each year to check for abnormalities in students' oral conditions (the number of decayed teeth, among other data) which might affect measurement.

#### 2.5.4. Food Intake

Participants' food intake was determined from a dietary record (recording food intake by portion size) completed by their guardians as surrogate respondents. The survey was conducted in July or August 2012 (summer vacation), during a three-day period extending over a holiday and weekdays. Special days, such as events and celebrations, were excluded. The recording sheets with guardian instructions were distributed and collected via the kindergartens.

The dietary record included all food and drink consumed by the participants between waking and going to bed. The dietary records collected were processed using the methods of the National Health and Nutrition Survey of Japan [24]. Dietary data were handled by an experienced registered dietician. The researchers examined completed dietary records. Nutrient values were calculated using the Standard Tables of Food Composition in Japan, 5th Revised and Enlarged Edition [25].

### 2.6. Sample Size and Power

An objective evaluation index was used to calculate sample size, with the mean value of the time-adjusted number of chews (528.0 chews per test meal) and the standard deviation (240.5 chews) obtained from previous cross-sectional studies [22]. The reason for this was the lack of any previous quantitative analysis of chewing habits in an intervention study format. We expected an increase of approximately 20% based on the mean (effect size) and 80% power, with significance established at *p* < .05. The sample size for the two-tailed test was 26 participants in each group (52 in total). However, because this study used multicluster rather than simple assignment, the sample size required might be larger (about +10–20 participants needed in each group). It was further expected that the yield rate of the effective data might be 65% (56–71 participants in each group), based on previous studies. Thus, the ideal sample size was determined to be 70 participants in each group (140 participants in total).

### 2.7. Statistical Analysis

Chewing habits were measured in all 150 participants. Response rates for each survey were as follows. The dietary record survey was completed by 51% (76 participants); the questionnaire survey pre- and post-intervention assessments were completed by 84% (126 participants) and 79.3% (119 participants), respectively. Physical state and oral condition were also investigated in all 150 participants.

Table 2 presents the number of responses and total eligible data received for each survey component. Dietary records containing three full days of data (32 from Group A and 38 from Group B, 70 participants total) and both pre- and post-intervention assessments were included in the analysis (56 from Group A and 52 from Group B, 108 participants total). No participants were excluded based on results from the oral checkups.

Some data were excluded under the following circumstances: participants measured once or less due to absence or dropout (32 participants); instances when the child could not finish the meal (15 participants); and data judged to be flawed due to sensitivity issues with the sensor (11 participants). In total, data from 96 participants were included in the final data analysis (50 from Group A and 46 from Group B).

We used the following equation to compare the time-adjusted number of chews as a rate of change index before and after the intervention: (the number after intervention − the number before intervention)/the number before intervention × 100 (%). Simple comparison between groups could not be used because the test meal contents varied between kindergartens.

For the obesity index, standard weight was first calculated using Murata's formula and coefficients for calculating standard weight-for-height [26]: for boys, standard weight [kg] = observed height [cm] × 0.386 − 23.699; for girls, standard weight [kg] = observed height [cm] × 0.377 − 22.750. Obesity index (%) was then calculated using the following formula: (weight [kg] − standard weight [kg])/standard weight [kg] × 100.

Energy and nutrient intakes were calculated using data from the 70 participants who completed three days' worth of dietary recording. The data of 108 participants who responded to all questions on the questionnaire both before and after the intervention were used for analysis. Given that this intervention was related to chewing habits, we selected two dimensions (chewing habits and chewing movement) in the questionnaire and then calculated a chewing habits score. The five degrees of each possible response were assigned a value, quantified in ascending order from least to greatest frequency:* never* = 5,* rarely* = 4,* sometimes* = 3,* frequently* = 2, and* always* = 1; the total score was therefore the sum of the values assigned to each answer.

The normality of the data was confirmed by the Shapiro-Wilk test, to determine the appropriate statistical analysis. Normally distributed data were analyzed using parametric methods; data not following a normal distribution were analyzed using nonparametric methods. For the nutrient intake data, nonparametric data for *β*-carotene and Vitamin B_1_ were used for analysis after logarithmic transformation. To compare differences in physical state, oral condition, and nutrient intake, two-tailed *t*-tests and Mann-Whitney *U* tests were completed. To examine the difference between the numbers of chews before and after intervention in each group, the Wilcoxon signed-rank test (two-tailed) was used. Nonpaired *t*-tests (two-tailed) were used to compare the difference between Groups A and B. The differences between measurements before and after the intervention were compared using an analysis of covariance designed to adjust for the baseline measurements. Data were analyzed using PASW Statistics Base 18 for Windows (SPSS Inc.); significance was set at 5% (*α* = .05). This study was approved by the Aomori University of Health and Welfare Ethics Review Committee (number 11054) consistent with the Declaration of Helsinki, 1989 revision. After obtaining authorization, written informed consent was obtained from the participants' guardians. To minimize the difference between the two groups, at the end of the intervention we distributed the paper textbook to Group B and asked its members to practice at home.

## 3. Results

### 3.1. Participants' Basic Characteristics and Dietary Intake

Table 3 shows the participants' basic characteristics and dietary intake. No significant differences were found in the participants' sex, age, height, weight, obesity index, number of decayed teeth, or energy and nutrient intake between the groups.

### 3.2. Evaluations

Table 4 shows the results of subjective and objective evaluations.

#### 3.2.1. Subjective Evaluation (Chewing Score by Questionnaire)

Subjective evaluation was based on the questionnaires completed by the children's guardians. The median chewing habits scores before and after the intervention were 29.0 and 31.0 in Group A and 28.5 points and 31.0 points in Group B. The scores showed a significant increase after the intervention in both groups (A, *p* = .040; B, *p* = .004). No significant difference was found in the changes between the groups (*p* = .597).

#### 3.2.2. Objective Evaluation (Chewing Habits Measured by the Sensor)

Objective evaluation was based on the indices of the direct chewing habits measurements. The median time-adjusted numbers of chews before and after the intervention were 520 and 628 in Group A and 498 and 634 in Group B. A significant increase was seen in the number of chews in both groups after the intervention: A, *p* = .000; B, *p* = .003. There was no significant difference in the median time-adjusted number of chews measured before the intervention. Moreover, there was no significant difference in the change between the groups (*p* = .523). The median rate of change in time-adjusted number of chews was +22.2% in Group A and +19.6% in Group B, which shows that the rate in Group A increased considerably but was not significant (*p* = .577). In Group B, the impact on the data which was caused by being a combined cluster was not different than Group A.

## 4. Discussion

In this study, we looked at the impact on chewing habits in young children of our nutritional education program to encourage children to “try to chew properly while enjoying eating.” We used two intervention approaches for the program, one with and one without practice at home. Contrary to our prediction, the results did not show a significant difference between program groups in changes in the children's chewing habits. However, the measurements before and after the interventions showed a significant difference for both program groups. We were able to quantitatively demonstrate for the first time the effect of a nutritional education program to encourage young children to try to chew properly.

### 4.1. Effect of the Educational Program

The reason children's attitude to try to chew was induced by this nutrition education program may be that its content was appropriate for the development stages of the 5-6-year-old subjects. Specifically, taking into account that, at that stage, self-sufficient, self-motivated eating is becoming possible as children are beginning to develop a positive attitude toward and interest/concern in eating and the sense of taste is developing, we designed the program content to include the following elements [27].

- A large picture book for storytelling was used to elicit the children's interest.
- Hand gestures were choreographed to go with the song to make singing it fun.
- A nutrition education paper notebook reinforced habits by providing pages for the children to make daily entries themselves.
- Content was integrated into the program to communicate the fun of eating using the five senses to get the children to feel that chewing properly when eating was “fun.”

These elements seem to be the reason why this educational program was able to influence children's attitudes to try to chew properly. Therefore, we propose that this program can promote good chewing habits effectively during the period when the body, mind, and senses grow, and children began to develop a positive attitude toward and interest/concern in eating.

### 4.2. Effects of the Educational Program Linking Guardians to an Institution

In Group A, the guardians were asked to assist so that the participants could complete the program at home in the same way as at kindergarten. Specifically, we offered information about nutrition education to the guardians and asked them to use the nutrition education paper textbook together with the participants. We hypothesized that additional engagement at home might lead to more significant modification of chewing habits in Group A than Group B; however, no such difference was found.

Consequently, we believe that the power of the additional intervention in Group A was inadequate, possibly due to the short intervention period; a difference between the groups may have been detected if the intervention period had been longer [28]. Furthermore, the teaching materials (the nutrition education paper textbook and the leaflet used in the additional intervention) were not prepared according to the stage of the guardians' habits modifications as proposed by the behavior modification model and the contents of that material provided only general messages. Therefore, the guardians may not have been sufficiently motivated to implement the educational program at home. Based on the obesity index distribution for all the participants, their obesity risk was small. Therefore, the guardians were less likely to be motivated toward obesity and lifestyle-related disease prevention for the participants.

On another front, influencing the guardians in Group B to some extent was unavoidable. Although the educational intervention for the guardians was not implemented in this group, we explained the study to the guardians and asked them to answer a self-report questionnaire; this may have influenced their responses. Consequently, this knowledge may promote similar changes in the guardians to those in Group A [29]. However, this remains unknown because evaluating changes in the guardians' consciousness and habits was outside the scope of this study.

We can also propose that the educational effect of the intervention, conducted similarly in Groups A and B in the classroom, was too large to detect any supplemental effect of the intervention added in Group A (even if such an effect existed). Another study on children belonging to the same age group reported that adequate educational effects were obtained only by the intervention performed at kindergarten, although the contents of this intervention were different from our study [30].

Although the additional guardian-led intervention in our study (encouraging the children at home) did not confer habitual effects, it is generally acknowledged that guardians' encouragement is important in early childhood [31]. Ideally, the kindergarten and guardians would collaborate on nutrition education in order to integrate and improve the learning effects obtained from the program [32].

### 4.3. Limitations

First, as to design limitations, the design was quasi-experimental because we were unable to establish a control (no intervention) group and we had to use cluster assignment for the experimental groups; individual participants in the same class could not be randomly assigned to different programs and the subjects could not be randomly assigned to programs due to the limited number of kindergartens. These limitations resulted in choosing kindergartens for the actual field settings. The annual schedule of education in each kindergarten led to difficulty in conducting the intervention at the same time.

It was also essential to complete a set of surveys and examinations for evaluation and to measure the number of chews even if no intervention (education) was performed. Requesting cooperation for data collection especially in the control group was a big challenge as in the general settings of educational interventions. Therefore, we could not set a complete control group without the intervention. There may also be various measurement biases derived from explanation to the guardians, entries in the questionnaire, and repeated measurement in the subjects. In addition, while all participants were subjected to the intervention itself, for the evaluation of the results we used a per-protocol approach and completed the analysis excluding individuals for whom we were not able to obtain valid data. Finally, low response rate (51% and 47%) for dietary assessment is another limitation. In this study, we did not find significant differences in the participants' energy and nutrient intake between the groups. Thus, it would be desirable if more responses were obtained, so that the differences between the groups could be clarified.

Notwithstanding these limitations and their related issues, in this study, we were able to demonstrate, for the first time through quantitative assessment, the impact of an educational program designed for introduction into nutrition education in kindergartens to encourage children to try to chew properly. The continued use of this program in these kindergartens may resolve childhood problems related to chewing and lead to formation of better chewing habits. It may also, hopefully, contribute to the enhancement of future health.

## 5. Conclusion

We compared the impact of two different intervention programs that promote trying to chew properly on children's chewing habits; one was conducted only in the kindergarten and the other involved the guardians. Although there was no difference in changes in the children's chewing habits between the programs, significant changes were observed before and after the intervention in both of these programs. Consequently, it was suggested that kindergarten school education alone to promote chewing food properly in early childhood was useful to establish good chewing habits.

## Figures and Tables

**Table 1:** Contents of the intervention for chewing well.

Contents	Frequency	Workout or not	
		Group A	Group B
Storytelling with large picture books	Once a week	*∗*	*∗*
Greetings before and after a meal	Every day	*∗∗*	*∗*
Singing a chorus with filksong and hand gesture	Three times a week	*∗∗*	*∗*
Chewing consciously while eating lunch	Once a week	*∗∗*‡	*∗*
Use of the nutrition education paper notebook^†^	Every day	*∗∗*	—
Providing information to encourage guardians to do the program at home	Once a week	*∗∗∗*	—

^*∗*^Performing in classroom.

^*∗∗*^Performing in classroom and at home.

^*∗∗∗*^Providing information to guardians.

^†^The nutrition education paper notebook designed for the study.

^‡^Checking chewing actions by the children and guardians.

**Table 2:** Number of responses and eligible data for each survey component.

	Collection, response, or measurement		Eligible data	Group A	Group B
*Data collection*					
Consented to participate	150		150	81	69
Physical status and oral conditions	150		150	81	69
The dietary record survey	76		70^*∗*^	32	38
Questionnaire survey	Before	126	108^†^	56	52
	After	119			
Chewing habits measurement	Before	135	96^‡^	50	46
	After	141			

Total number of subjects: *N* = 150.

^*∗*^Dietary records completed for three days.

^†^Questionnaires collected both before and after intervention.

^‡^Data measured once or less due to absence or study dropout (*n* = 32).

Child did not complete the meal, at least once (*n* = 15).

Data judged to be problematic because of poor sensor sensitivity (*n* = 11).

**Table 3:** Basic characteristics and dietary intake of 150 Japanese preschool children aged 5-6 years.

	Group A (*n* = 81)	Group B (*n* = 69)	*p* value
Sex			
Boys	39	32	.958^‡^
Girls	42	37	
Age^*∗*^			
<6 years	50	43	.941^‡^
>6 years	31	26	
Physical status and oral condition			
Body height (cm)			
Boys	111.5 ± 5.4	111.3 ± 3.9	.869^§^
Girls	111.4 ± 5.6	110.7 ± 4.8	.526^§^
Body weight (kg)			
Boys	18.8 [12.9–33.8]	18.4 [15.2–27.8]	.986^‖^
Girls	18.6 [13.5–25.7]	18.4 [13.2–29.0]	.840^‖^
Obesity index^†^ (%)			
Boys	−4.0 [−14.0–39.0]	−3.0 [−13.0–31.0]	.746^‖^
Girls	−4.0 [−28.0–21.0]	−3.0 [−17.0–41.0]	.619^‖^
Number of untreated teeth			
Boys	.0 [0–12]	.0 [0–8]	.194^‖^
Girls	.0 [0–9]	.0 [0–10]	.554^‖^
Energy and nutrient intake^‡^	*n* = 32	*n* = 38	
Energy (kcal)	1426 ± 217	1352 ± 257	.203
Protein (g)	46.7 ± 8.8	44.7 ± 10.3	.386
Fat (g)	44.6 ± 10.8	43.7 ± 13.1	.762
Carbohydrate (g)	205.8 ± 37.1	191.0 ± 32.4	.079
Calcium (mg)	421 ± 177	366 ± 133	.141
Iron (mg)	4.7 ± 1.5	4.6 ± 1.2	.750
*β*-carotene (*μ*g)	1973 ± 1186	2060 ± 1549	.804
Vitamin B1 (mg)	0.65 ± 0.20	0.58 ± 0.16	.156
Vitamin B2 (mg)	0.85 ± 0.26	0.74 ± 0.24	.084
Vitamin C (mg)	63 ± 33	56 ± 266	.329
Folate (*μ*g)	172 ± 67	159 ± 47	.307
Dietary fiber (g)	9.4 ± 3.7	8.7 ± 2.6	.336
Sodium (g)	6.8 ± 1.8	6.6 ± 2.0	.762

Body height: mean ± SD (standard deviation); body weight and obesity index; untreated teeth: median [range].

Energy and nutrient intake: mean ± SD (standard deviation score). Only *β*-carotene and Vitamin B1: values after logarithmic transformation were used for comparison between groups.

^*∗*^The start of this intervention.

^†^Calculated according to the Murata method.

^‡^Chi-square-test, ^§^ *t*-test (two-tailed), ^‖^Mann-Whitney *U* test (two-tailed), and ^†^nonpaired *t*-test (two-tailed).

**Table 4:** Chewing habits before and after the intervention.

	Group A			Group B			*p* value^§^
	Before	After	*p* value^‡^	Before	After	*p* value^‡^	
*Subjective evaluation*	(*n* = 81)			(*n* = 69)			
Chewing habits score (points)	29.0 [25.0–32.0]^a^	31.0 [24.5–33.0]^a^	.040	28.5 [23.0–32.0]^b^	31.0 [25.0–34.0]^b^	.004	.597^††^
*Objective evaluation*							
Time-adjusted number of chews (times)	520 [407–713]^c^	628 [531–808]^c^	.000	498 [421–700]^d^	634 [498–854]^d^	.003	.523^††^
Rate of change (%)	22.2 [3.0–47.1]			19.6 [−5.2–57.4]			.577
The number of chews (times)	523 [406–714]	612 [449–783]	.001	474 [363–704]	683 [450–818]	.000	
Meal time (min)	29.5 [21.9–37.5]	25.5 [19.5–31.3]	.002	22.3 [17.5–33.1]	23.5 [19.5–32.1]	.635	

Median [25–75%]; ^a^ *n* = 56; ^b^ *n* = 52; ^c^ *n* = 50; ^d^ *n* = 46.

*Definitions*

The score of chewing habits: total score of seven questionnaire items related to chewing habits and chewing movement.

Time-adjusted number of chews: to consider the influence of meal time by the residual method.

Rate of change: in the comparison of the time-adjusted number of chews, before and after intervention.

Meal time: duration of time spent eating the test meal.

The number of chews: the number of times participants chewed during a test meal.

Meal times: from start to end.

^‡^Wilcoxon signed-rank test (two-tailed).

^§^Nonpaired *t*-test (two-tailed).

^††^Analysis of covariance (adjusted with the baseline measurements).
